# Primary ectopic breast cancer of the vulva, treated with local excision of the vulva and sentinel lymph node biopsy: a case report

**DOI:** 10.1186/s40792-017-0343-x

**Published:** 2017-05-16

**Authors:** Takayuki Ishigaki, Yasuo Toriumi, Ryouko Nosaka, Rei Kudou, Yoshimi Imawari, Makiko Kamio, Hiroko Nogi, Hisashi Shioya, Hiroshi Takeyama

**Affiliations:** Department of Breast and Endocrine Surgery, The Jikei University School of Medicine, 3-25-8, Nishi-shinbashi, Minato-ku, Tokyo, 105-8461 Japan

**Keywords:** Ectopic breast cancer, Breast cancer of the vulva, Sentinel lymph node biopsy

## Abstract

Primary breast cancer fairly infrequently occurs in ectopic breast tissue, and primary ectopic breast cancer of the vulva is particularly rare. Only 26 cases have been published in the English-language literature, and there has been no report of primary breast carcinoma of the vulva in Japan. We report a rare case of primary ectopic breast cancer of the vulva that was treated with local excision of the vulva and sentinel lymph node biopsy (SLNB). The patient was a 72-year-old woman who had noticed a right vulvar tumor 10 years earlier. The tumor was excised by the Department of Plastic Surgery of our hospital. The histology of the vulvar tumor revealed an invasive ductal carcinoma of the breast, and immunohistochemical staining of the vulvar specimen showed the tumor cells to be 100% estrogen-receptor-positive and 100% progesterone-receptor-positive. All margins of resection were positive for neoplastic involvement. An additional local excision of the vulva and right inguinal SLNB were performed in our department. The intraoperative frozen section was negative for metastasis, and lymph node dissection was not performed. The final pathology was negative for residual disease, and a partially normal ductal component was present. Adjuvant hormonal therapy with an aromatase inhibitor was indicated post-operatively. The patient was asymptomatic and free of detectable disease at a 6-month follow-up. Due to the rarity of this diagnosis, there are no established guidelines for treatment. Although cases in which SLNB was performed are rare, we consider SLNB to be an effective alternative to inguinal node dissection for ectopic primary breast cancer of the vulva.

## Background

Ectopic mammary tissue may occur anywhere along the milk line, which extends bilaterally from the axilla to the groin. The frequency of ectopic breast tissue in females is 1 to 6%, and it is relatively common in the axilla or on the thorax but rare in the vulva [1]. Primary breast cancer fairly infrequently occurs in the ectopic breast tissue, and ectopic primary breast cancer of the vulva is particularly rare. Only 26 cases have been published in the English-language literature. There are no guidelines for treating breast cancer of the vulva because of its rarity. We report a rare case of primary ectopic breast cancer of the vulva that was treated with local excision of the vulva and sentinel lymph node biopsy (SLNB).

## Case presentation

**Fig. 1:**
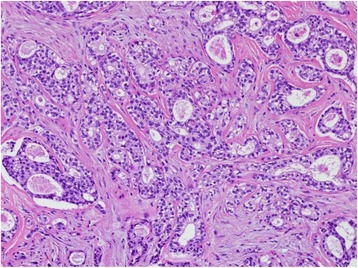
Histopathological findings of the resected specimen (hematoxylin-eosin stain, ×200). Invasion of the stroma by ductal adenocarcinoma

**Fig. 2:**
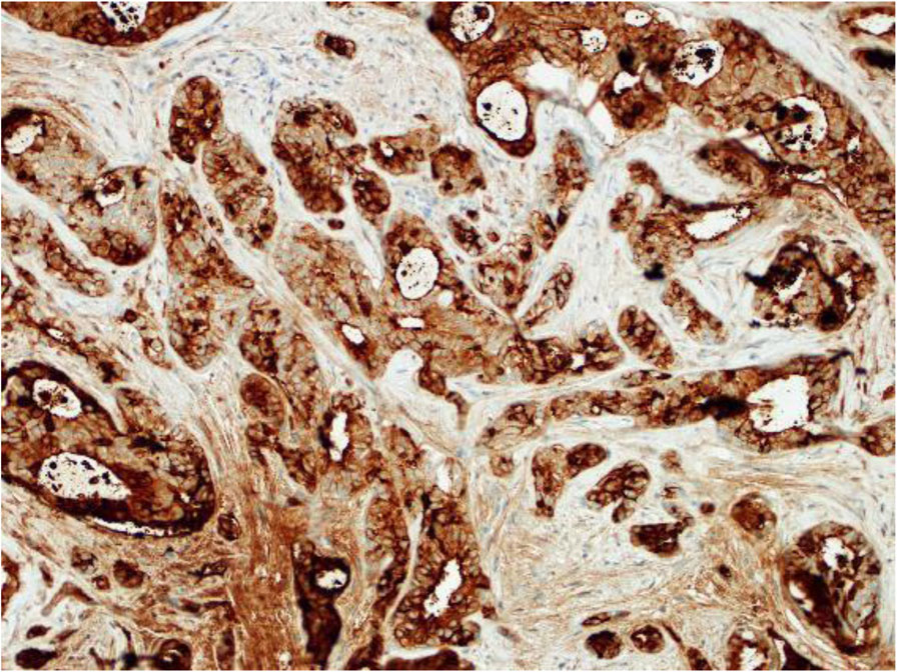
Histopathological findings of the resected specimen (GCDFP-15 stain, ×200). Positive for GCDFP-15 expression

The patient was a 72-year-old woman (gravida 0 and para 0), with no history of malignancy or breast disease and no family history of carcinoma. She had been aware of a left third finger tumor for 20 years and a right vulvar tumor for 10 years. These tumors were excised at the Department of Plastic Surgery of our hospital. The histology of the finger tumor revealed a schwannoma, and the vulvar tumor revealed an invasive ductal carcinoma of the breast (see Fig. 1). Immunohistochemical staining of the vulvar specimen showed the tumor cells to be 100% estrogen-receptor-positive, 100% progesterone-receptor-positive, human epidermal growth factor 2-negative, and gross cystic disease fluid protein 15 (GCDFP-15)-positive (see Fig. 2). All margins of resection were positive for neoplastic involvement. The patient was referred to our department for further assessment and treatment. There was a 20-mm operation scar in the upper right direction of the right labia majus. The results of the breast examination and mammary imaging (mammography and ultrasound) were negative for primary breast tumors. The chest-abdomen-pelvic CT scan, bone scintigraphy, and inguinal ultrasound showed no secondary neoplastic lesions. The diagnosis of a primary ectopic breast cancer of the vulva was established.

**Fig. 3:**
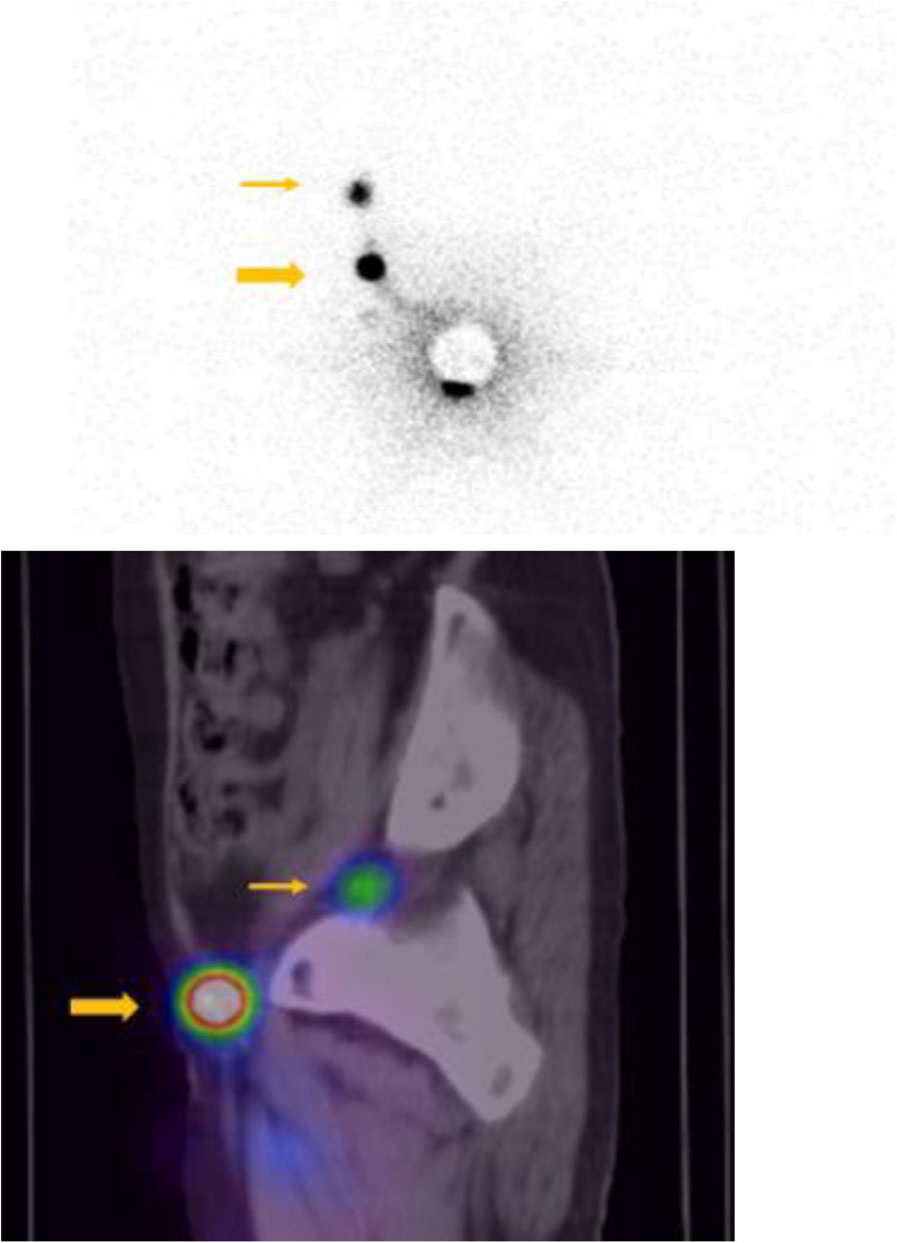
Lymphoscintigraphy showed hot areas at the right inguinal lymph node (*thick arrow*) and right obturator lymph node (*thin arrow*)

**Fig. 4:**
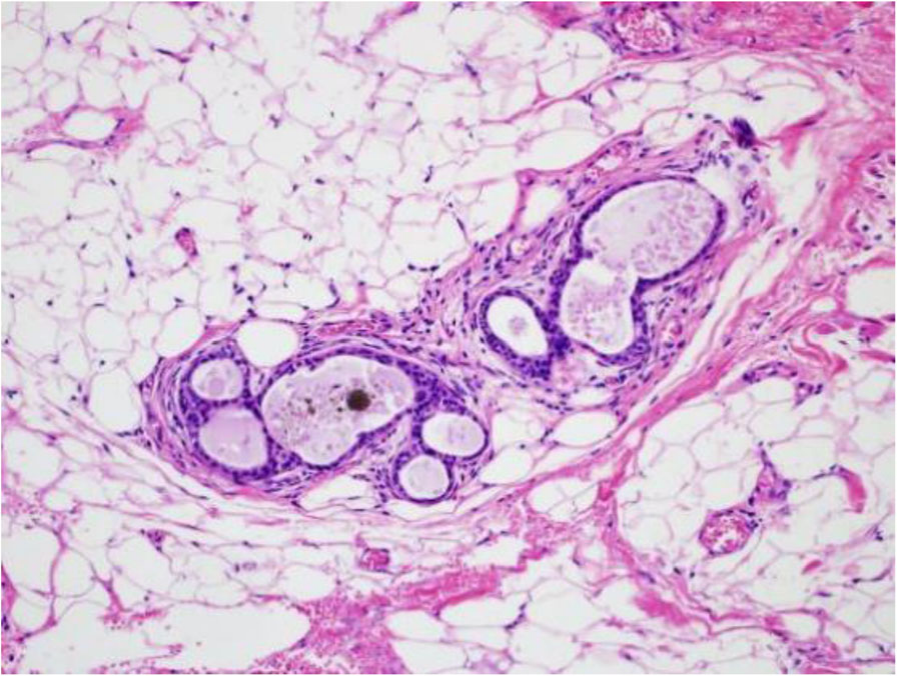
Histopathological findings of the additional specimen (hematoxylin-eosin stain, ×200). A normal mammary ductal component was present

Additional local excision of the vulva and SLNB were performed. We used preoperative ^99m^Tc-phytic acid lymphoscintigraphy and indocyanine green (ICG) lymph node localization in the SLNB. ^99m^Tc-phytic acid was injected into the subcutaneous tissue near the previous right surgical site on the day before surgery. Lymphoscintigraphy showed hot areas at the right inguinal lymph node and right obturator lymph node (see Fig. 3). The inguinal lymph node was determined to be a sentinel lymph node based on the image. In the operation, ICG was also injected into the same site near the previous operative scar. A right inguinal incision was created, and the γ-probe was used to identify the hot area. A hot, green sentinel lymph node was identified and excised. Then, an additional local excision of the vulva with 10-mm margins from the previous operative scar was performed. The intraoperative frozen section was negative for sentinel lymph metastasis, and a lymph node dissection was not performed. The final pathology was negative for residual disease, and a partially normal ductal component was present (see Fig. 4). Adjuvant hormonal therapy with an aromatase inhibitor was indicated post-operatively. The patient was asymptomatic and free of detectable disease at a 6-month follow-up.

### Discussion

Ectopic breast tissue can occur anywhere along the primitive embryonic milk lines and may develop benign and malignant pathologic processes similar to those seen in normally located breast tissue, such as fibroadenoma, intraductal papilloma, fibrocystic disease, lactating adenoma, hidradenoma papilliferum, and carcinoma [2, 3]. Any of the various histological subtypes of breast cancer may occur in the vulva, including infiltrating ductal, lobular, mucinous, mixed ductal, and lobular carcinomas [3, 4]. There have been 26 cases of primary ectopic breast carcinoma of the vulva described in the English-language literature since the first report by Green in 1935 (see Table 1). Our case is the first reported case of primary breast carcinoma of the vulva in Japan and the 27th case reported worldwide.

**Table 1:** The characteristics of primary ectopic breast carcinoma of the vulva

Ref.	Year	Age	Size (cm) operation		Adjuvant therapy	Histology	ER	PR	Her2	LN	Status	Follow-up (months)
Greene [12]	1935	59	20	None	None	Adenocarcinoma	*	*	*	*	Dead	1
Hendrix [13]	1956	58	3	Vulvectomy	None	Adenocarcinoma	*	*	*	*	Dead	4
Guerry [14]	1976	62	1.5	Partial	None	Ductal carcinoma	*	*	*	*	Dead	24
Cho [15]	1985	70	4	Hemi-vulvectomy + LND	Tamoxifen	Adenocarcinoma	(+)	(+)	*	2/9	DF	24
Simon [16]	1988	60	2	Vulvectomy + LND1	CT + tamoxifen	Adenocarcinoma	(+)	(+)	*	3/11	Dead	27
Rose [17]	1990	68	3.5	Vulvectomy	RT + tamoxifen	Ductal carcinoma	(+)	(−)	*	1/15	*	*
Bonito [18]	1992	46	1.5	Vulvectomy + LND	None	*	*	*	*	11/13	DF	4
Bailey [19]	1993	65	3	Vulvectomy + LND	Tamoxifen	Ductal carcinoma	(+)	(+)	*	2/20	DF	12
Levin [20]	1994	62	2.5	Partial + LND	Tamoxifen	Adenocarcinoma	(+)	(−)	(+)	4/11	DF	24
Kennedy [21]	1997	71	5	Vulvectomy + LND	CT + RT	*	(−)	(−)	*	9/9	DF	15
Irvin [22]	1998	64	3	Partial + LND	CT + RT-F tamoxifen	Adenocarcinoma	(+)	(+)	*	1/14	DF	4
Gorisek [23]	2000	81	3	Partial	Tamoxifen	Adenocarcinoma	(+)	(+)	*		DF	19
Neumann [24] 2000		60	3	Hemi-vulvectomy + LND	CT + RT + tamoxifen	ILC	(+)	(+)	*	21/21	DF	20
Piura [25]	2002	69	3	Vulvectomy + LND	CT + tamoxifen	Adenocarcinoma	(+)	(+)	*	7/15	DF	14
Chung [26]	2002	47	2	Vulvectomy	None	Mucinous	(+)	(+)	(−)	*	DF	36
Yin [27]	2003	84	5	Partial + LND	None	Mucinous	(+)	(+)	(−)	1/11	DF	9
Lopes [28]	2006	44	2	Partial + LND	CT + tamoxifen	Mucinous	(+)	*	(−)	2/13	*	*
Fracchioli [29] 2006		57	1	Vulvectomy + LND	CT + tamoxifen	Adenocarcinoma	(−)	*	*	7/7	Rec	36
North [4]	2006	49	1.5	Partial + LND	CT + tamoxifen	Ductal carcinoma	(+)	(+)	(−)	5/7	*	*
Martinez [10]	2007	49	3.5	Partial + SLNB—LND	Tamoxifen	Ductal carcinoma	(+)	(+)	*	0/14	*	*
Naseer [30]	2011	57	1.5	Partial + LND	CT + aromatase	Ductal carcinoma	(+)	(+)	(−)	3/13	*	*
Diniz [2]	2012	82	2	Partial	RT + letrozole	IDC	(+)	(+)	*	*	DF	48
McMaster [31] 2013		60	3	Partial	RT	Ductal carcinoma	(+)	*	*	*	*	*
Bogani [9]	2013	71	4	Vulvectomy + SLNB—LND	CT + tamoxifen	Ductal carcinoma	(+)	(+)	*	1/8	DF	24
Lamb [5]	2013	59	1	Partial + LND	Tamoxifen	Adenocarcinoma	(+)	(+)	(−)	*	*	*
James [7]	2015	62	1.3	Partial	CT + RT	IDC	(+)	(+)	(−)	*	Rec	13
Present case	2016	72	1.5	Partial + SLNB	Aromatase	IDC	(+)	(+)	(−)	0/1	DF	6

*LND* (inguinal)lymph node dissection, *LND1* (inguinal and pelvic) LND, *SLNB* sentinel lymph node biopsy, *CT* chemotherapy, *RT* radiotherapy, *ILC* invasive lobular carcinoma, *IDC* invasive ductal carcinoma, *Dead* death of disease, *DF* disease-free, *Rec* recurrence, * unknown

A diagnosis of primary ectopic breast cancer located in the vulva has generally been based on histopathologic patterns. Histology criteria for the diagnosis include (1) a morphology consistent with breast carcinoma; (2) positive estrogen and/or progesterone-receptor expression on immunohistochemical staining; (3) immunostaining positive for additional common breast cancer-associated markers including carcinoembryonic antigen, CK7, and mammoglobin; and (4) presence of a non-neoplastic breast tissue or carcinoma in situ component [5]. The GCDFP-15 used in our case is also a specific marker of breast cancer [6]. In addition, it is necessary to exclude metastasis from primary orthotopic breast carcinoma or adenocarcinoma of other organs.

Sentinel node mapping in vulva cancer is a more contemporary topic in the literature [7]. An observational study followed 403 patients with primary vulvar cancer of less than 4 cm that had been treated with sentinel node mapping. In 276 patients with vulvar disease and a negative sentinel node (median follow-up time, 35 months), eight inguinal recurrences were diagnosed (3.0%). The inguinal recurrence rate was low; therefore, it was suggested that sentinel node dissection, performed by a quality-controlled multidisciplinary team, should be part of the standard treatment in selected patients with early-stage vulvar cancer [8]. In most cases, surgical methods for primary ectopic breast cancer of the vulva included surgical excision with inguinal lymph node dissection. One published study utilized sentinel lymph node biopsy for recurrent case after a previous lymph node dissection [7]. Two studies reported the use of SLNB for non-recurrent cases followed by complete ipsilateral inguinal lymph node dissection [9, 10]. Our case is the first reported case of only SLNB for non-recurrent primary breast cancer of the vulva. We identified the sentinel lymph node in ipsilateral inguinal lymph nodes as effectively as other reports in which isotope or dye used.

We suppose that pelvic node resection following inguinal lymph node resection is necessary if the sentinel node is found to be positive for ectopic breast cancer of the vulva because, in our case, lymphoscintigraphy showed a hot area in a right obturator lymph node. In a randomized, controlled trial that followed 114 patients with vulvar cancer with groin node metastasis allocated to postoperative pelvic and groin radiation or to ipsilateral pelvic node resection after radical vulvectomy and inguinal lymphadenectomy, long-term follow-up confirmed a significant recurrence-free and cancer-related death benefit of radiation compared with pelvic node resection [11]. In the case of the primary ectopic breast cancer of the vulva, pelvic node resection and/or pelvic and groin radiation should (after ipsilateral inguinal lymph node dissection) be considered if the inguinal lymph node is positive.

Due to the rarity of this diagnosis, there are no established guidelines for postoperative treatment. Virtually all of the literature consists of individual case reports, and most authors recommend appropriate treatment for primary orthotopic breast cancer of a similar stage. Therefore, treatment should consist of an individualized combination of surgery, chemotherapy, trastuzumab therapy, radiotherapy, and hormonal therapy [4].

## Conclusions

Ectopic primary breast cancer of the vulva is an extremely rare condition, and diagnosis is made based on the pathology as well as exclusion of orthotopic breast cancer. Due to the rarity of this diagnosis, there are no established guidelines for the treatment of the patient. The appropriate treatment for a primary orthotopic breast cancer of a similar stage is recommended. Our patient was treated with local excision of the vulva and SLNB and adjuvant hormonal therapy with an aromatase inhibitor. We consider SLNB to be an effective alternative to inguinal node dissection for ectopic primary breast cancer of the vulva.

## Abbreviations

GCDFP-15: Gross cystic disease fluid protein 15
ICG: Indocyanine green
SLNB: Sentinel lymph node biopsy
